# Defining frequent attendance in general practice

**DOI:** 10.1186/1471-2296-9-21

**Published:** 2008-04-15

**Authors:** Frans TM Smits, Jacob J Mohrs, Ellen E Beem, Patrick JE Bindels, Henk CPM van Weert

**Affiliations:** 1 Department of General Practice, Academic Medical Centre, University of Amsterdam, PO Box 22700, 1100 DE, Amsterdam, The Netherlands; 2 Julius Centre for Health Sciences and Primary Care, University Medical Centre Utrecht, PO Box 85500, 3508 Utrecht, The Netherlands; 3 Medical Health Centre Reigersbos, Ravenswaaipad 110, 1107EG, Amsterdam, The Netherlands

## Abstract

**Background:**

General practitioners (GPs) or researchers sometimes need to identify frequent attenders (FAs) in order to screen them for unidentified problems and to test specific interventions. We wanted to assess different methods for selecting FAs to identify the most feasible and effective one for use in a general (group) practice.

**Methods:**

In the second Dutch National Survey of General Practice, data were collected on 375 899 persons registered with 104 practices. Frequent attendance is defined as the top 3% and 10% of enlisted patients in each one-year age-sex group measured during the study year. We used these two selections as our reference standard. We also selected the top 3% and 10% FAs (90 and 97 percentile) based on four selection methods of diminishing preciseness. We compared the test characteristics of these four methods.

**Results:**

Of all enlisted patients, 24 % did not consult the practice during the study year. The mean number of contacts in the top 10% FAs increased in men from 5.8 (age 15–24 years) to 17.5 (age 64–75 years) and in women from 9.7 to 19.8. In the top 3% of FAs, contacts increased in men from 9.2 to 24.5 and in women from 14 to 27.8.

The selection of FAs becomes more precise when smaller age classes are used. All selection methods show acceptable results (kappa 0.849 – 0.942) except the three group method.

**Conclusion:**

To correctly identify frequent attenders in general practice, we recommend dividing patients into at least three age groups per sex.

## Background

In primary care, the workload of General Practitioners (GPs) is significantly related to a minority of patients who consult more frequently than their peers [1]. Studies are consistent in confirming that these frequent attenders (FAs) have high rates of physical disease, psychiatric illness, social difficulties and emotional distress [2-4]. Because frequent attendance can be related to undisclosed medical problems, identifying FAs could help GPs to select those patients who may need an adjustment to the care they receive [5]. The combination of large workload and high rate of (chronic) disease make FAs an important group for a GP not only to study but also to treat. Exceptional attendance is also considered as an indicator of inappropriate consulting behaviour and healthcare use [6-11]. Health services research has therefore used frequent attendance for identifying both inadequate health care delivery and possible misuse of health care. Trials on the effect of (mainly psychiatric) interventions on the attendance rate and morbidity of FAs showed conflicting results [12-15]. In a review of interventions on FAs we found indications that frequent attendance might be a sign of as yet undiagnosed major depressive disorder (MDD) and that treatment of MDD might improve the depressive symptoms and the quality of life of depressed FAs. We found no evidence that it is possible to influence healthcare utilization [13,15-17].

The interpretation of studies on frequent attendance is hampered because of differences in the organisation of health care, the setting and the definition of FAs. Age and sex have been shown to be highly associated with the frequency of attendance [18]. Selecting FAs without adjusting for age and sex will predominantly result in the selection of older women [19]. Therefore, any study of frequent attendance requires a clear definition of these patients and a clear description of the selection process. After reviewing the literature on frequent attendance, Vedsted suggested that frequent attendance should be defined as a proportional part (highest 10%) of all attenders, stratified for age and sex [20].

Selecting FAs by using age groups with a small band (for instance ten-year groupings) is difficult, especially in smaller populations like those of a (group) practice because of the resulting low number of patients in each cell. Therefore, Howe et al. developed an easy cohort definition to identify those patients whose attendance patterns are unusual for their sex and age. She stated that, dividing the male population into two different age groups (15–44; 45–74 years), would result in including 95% of the total male patients identified as attending at or above the 97th percentile compared with the more complex procedure of ten-year groupings. Further, she concluded that no such division of the population was needed for females, as their consultation rates were considered fairly constant. She advised further analysis on the validity of this method in other populations to be performed [21].

In the Netherlands every citizen is enlisted by one GP and Dutch inhabitants consult their own GP for all medical complaints. The GP functions as a gatekeeper for specialist care. GP-care in 2001 was paid either by a social sick-fund or an obligatory private insurance. We used the large database of the second Dutch National Survey of General Practice (2001) as a unique possibility for comparing the quality of different FA selection methods in general practice in the Netherlands. Our aim was to assess these methods and to identify the most feasible and effective one for use in an average general (group) practice.

## Method

**Figure 1:**
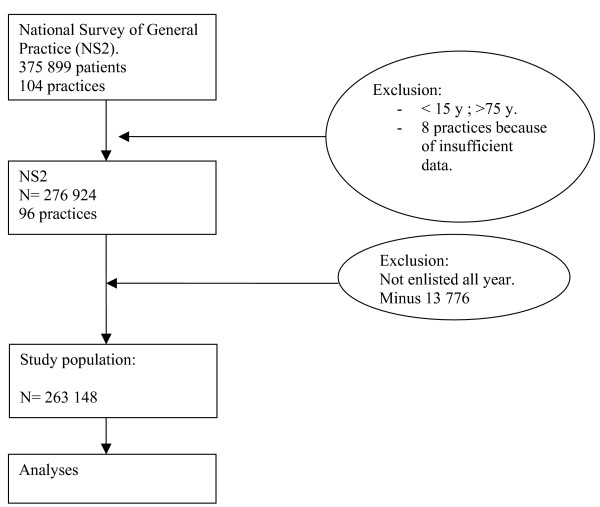
Flow diagram of included patients and practices.

In the second Dutch National survey of General Practice, data were collected over a one-year period on health and healthcare-related behaviour from 375 899 persons, registered with 104 practices. Eight practices were excluded because of insufficient data (see Fig 1). Population, practices and GPs were representative for the Dutch population, with a slight under-representation of single-handed GPs. The study design, methods, response and quality of the data of this extensive second Dutch National Survey have been published elsewhere in more detail [22-24]. To correct for loss or growth of the practices involved during the study year, we used the data of patients enlisted within each practice over the complete one-year period (n = 263 148) as the denominator. As most previous studies on frequent attendance have excluded children and the very old, we also only used the data of patients between the ages of 15 and 74 years.

For all patients included, each contact with the primary care team (consultations, house calls and telephone calls) was registered. We calculated the contact frequencies of all patients between the ages of 15–74 years for every combination of age and sex. As in previous research, the top 3% and the top 10% consulting patients from this calculation were defined as FAs. We also included patients with no attendance. These two selections were then used as our reference standard.

As index-selections, we selected the top 3% and 10% of FAs of the same population by dividing the genders into four different age group clusters ranging from just one to as many as six. We compared the sensitivity and specificity of the selection criteria in each of these four cluster groups:

1) Per each 10-year age band: 6 classes per sex category [25,26].

2) Per each 15-year age band: 4 classes per sex category.

3) According to the sex-age grouping, used by the WONCA classification committee, we tested an adjusted selection method with 3 age classes per sex category: 15–44 years, 45–64 years and 65–74 years [27].

4) Dividing males into two separate cohorts (15–44 years; 45–74 years) and all women in one cohort: the three group method [28].

By constructing four by four tables we calculated the test characteristics (the sensitivity, specificity, positive and negative predictive value and kappa) of these four clusters, using the one-year age band method as reference standard. All data were analysed using SPSS 14.0 for windows.

The study was conducted according to the Dutch legislation on data protection (Ministry of Justice, the Netherlands)

## Results

**Figure 2:**
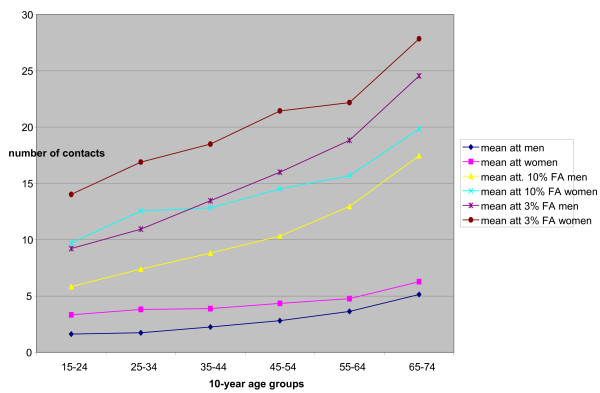
Mean attendance per sex: all attenders and the top 3%/10% frequent attenders.

From the total number of enlisted patients, 63102 (24%) of which 21 090 (16%) female and 42 012 (32%) men did not consult their primary care practice during the study year. Women consulted more frequently than men and older age correlated with a rising number of contacts for both sexes (Fig 2). The mean number of contacts increased in men from 1.62 (age 15–24 years) to 5.13 (age 65–74 years) and in women from 3.32 (age 14–24 years) to 6.27 (65–74 years). The mean attendance by sex and age of the top 3% and 10% attenders is presented in Fig 2. The mean number of contacts in the top 10% FAs increased in men from 5.84 (age 15–24 years) to 17.46 (age 64–75 years) and in women from 9.72 (age 15–24 years) to 19.83 (age 64–75 years). The mean number of contacts in the top 3% of FAs increased in men from 9.21 (age 15–24 years) to 24.52 (age 64–75 years) and in women from 14.02 (age 15–24 years) to 27.83 (age 64–75 years).

All test characteristics (sensitivity, specificity, positive and negative predictive value and kappa), are summarized in Table 1. With 6 classes, the kappa is 0.942 (10 % FA) and 0.925 (3% FA) but with the 3 group-method the kappa is 0.818 (10% FA) and 0.756 (3% FA). Test characteristics improve with smaller age classes and logically sensitivity drops by using the three group method, even more in females than in males. The test characteristics are slightly better when the top 10 % is selected instead of the top 3% of FAs. All methods show acceptable results (kappa 0.849 – 0.942) except the three group method.

**Table 1:** Overview of the test characteristics of the four selection methods.

	6 cl. method 3%FA^1^		6 cl. method 10%FA^2^		4 cl. method 3%FA^3^		4 cl. method 10%FA^4^	
	women	men	women	men	women	men	women	men
Sensitivity	94,2	89,4	94,0	93.1	89.4	93.6	94.7	91.5
Specificity	99,8	99,8	99,3	99.8	99.8	99.6	99.6	99.1
PPV^5^	93,7	93,7	94,3	98.2	93.7	89.5	96.3	93.1
NPV^6^	99,8	99,6	99,2	99.0	99.6	99.8	99.3	98.8
Kappa	0.937	0.912	0.934	0.950	0.912	0.911	0.949	0.912
								
	3 cl. method 3%FA^7^		3 cl. method 10%FA^8^		3 group method 3%FA^9^		3 group method 10%FA^10^	
	women	men	women	men	women	men	women	men
Sensitivity	89.0	84.8	96.0	85.5	76.1	73.6	79.3	86.5
Specificity	99.3	99.3	98.3	98.8	99.1	99.4	98.5	97.6
PPV	88.1	80.4	87.8	90.6	74.1	82.3	86.9	83.1
NPV	99.6	99.5	99.5	98.0	99.2	99.1	97.4	98.1
Kappa	0.881	0.819	0.906	0.864	0.742	0.770	0.809	0.826
								
	6 cl. method		4 cl. method		3 cl. method		3 group method	
	3% FA	10% FA	3% FA	10% FA	3% FA	10% FA	3% FA	10% FA
	Men and women		Men and Women		Men and women		Men and Women	
Sensitivity	91.8	93.5	90.9	93.0	86.9	90.6	74.8	83.0
Specificity	99.8	99.5	99.7	99.3	99.4	98.5	99.3	98.0
PPV	93.7	96.3	91.4	94.6	84.1	89.1	78.0	84.8
NPV	99.7	99.1	99.7	99.1	99.5	98.7	99.1	97.7
Kappa	0.925	0.942	0.912	0.930	0.849	0.885	0.756	0.818

1. 6 cl. method: selection FAs per 10 years of age.

2. Idem

3. 4 cl. method: selection of FAs per 15 years of age

4. Idem

5. positive predictive value

6. negative predictive value

7. 3 cl. method: selection of FAs in 3 age groups (15–44; 45–64; 65–74).

8. Idem

9. 3 group method: selection of male FAs in two age groups (15–44; 45–74) and women in one group.

10. Idem

## Discussion

The purpose of this study was to compare different methods for selecting frequent attenders in primary care and to identify the most feasible method with acceptable test characteristics in a general (group) practice. We found specificity to be about the same in all methods, but sensitivity diminishes gradually when larger age groupings are used and shows a drop in the three group method. This means that with the three group method (3% resp.10% FA) 25% resp. 17 % of the FAs will not be identified. For instance selecting the top 10% of FAs the three group method misses 5247 FAs (17%) of which 58% female and 47% in the age between 15 and 24.

This study is the first attempt to compare different methods of identifying FAs. In a large database like the Dutch National Survey, the reference method (with one year sex-age bands) is the most precise method for identifying FAs. In smaller databases however, such a method results in very few patients within each age band and is therefore not feasible. Our results demonstrate that specificity and sensitivity for identifying FAs increases when smaller age groups are used, as could be expected. On the level of a general (group) practice, less precise methods can be used with acceptable results: for instance, by dividing all patients into at least 3 age cohorts per sex. For studies on larger patient groups, it is best to use the smallest possible age groupings, mainly for reasons of positive predictive value. Standardisation of methods for selecting FAs is needed in order to allow comparisons between studies to take place.

The purpose for which FAs need to be selected, as well as the limitations of the database, can determine the degree of the desired precision. For example, if a GP wants to use frequent attending as a red flag pointing at unidentified medical problems, it would not be too big a problem to incorrectly select a patient (false positive). Not selecting an FA (false negative) seems to be a bigger problem, but the negative predictive value is high in all methods. However researchers have to use the smallest age band possible to correctly select FAs.

## Conclusion

We conclude that in order to identify exceptional users of health care, sex and age have to be taken into account. The best method for identifying frequent attenders is to use small age and sex groups. If this is not possible or needed, for instance in a single general (group) practice, we recommend that GPs divide their patients into at least 3 age groups per sex category in order to identify their exceptional attenders.

## Competing interests

The author(s) declare that they have no competing interests.

## Authors' contributions

FS conceived the study, participated in its design and coordination and drafted the manuscript. JM participated in the design of the study and performed the statistical analysis. EB participated in the design of the study. PB participated in the design and helped to draft the manuscript. HvW helped to conceive the study, participated in its design and helped to draft the manuscript. All authors read, commented upon and approved the final manuscript.

## Pre-publication history

The pre-publication history for this paper can be accessed here:


